# Transdermal Nicotine Application Attenuates Cardiac Dysfunction after Severe Thermal Injury

**DOI:** 10.1155/2015/292076

**Published:** 2015-07-28

**Authors:** Leif Claassen, Stephan Papst, Kerstin Reimers, Christina Stukenborg-Colsman, Lars Steinstraesser, Peter M. Vogt, Theresia Kraft, Andreas D. Niederbichler

**Affiliations:** ^1^Department of Orthopedics, Hannover Medical School, 30625 Hannover, Germany; ^2^Department of Anesthesiology, Hannover Medical School, 30625 Hannover, Germany; ^3^Department of Plastic, Hand and Reconstructive Surgery, Hannover Medical School, 30625 Hannover, Germany; ^4^Department of Plastic, Hand and Reconstructive Surgery, Evangelical Hospital Oldenburg, 26122 Oldenburg, Germany; ^5^Department of Molecular and Cell Physiology, Hannover Medical School, 30625 Hannover, Germany; ^6^Department of Hand and Plastic Surgery, Helios Klinikum Berlin-Buch, 13125 Berlin, Germany

## Abstract

*Background*. Severe burn trauma leads to an immediate and strong inflammatory response inciting cardiac dysfunction that is associated with high morbidity and mortality. The aim of this study was to determine whether transdermal application of nicotine could influence the burn-induced cardiac dysfunction via its known immunomodulatory effects. *Material and Methods*. A standardized rat burn model was used in 35 male Sprague Dawley rats. The experimental animals were divided into a control group, a burn trauma group, a burn trauma group with additional nicotine treatment, and a sham group with five experimental animals per group. The latter two groups received nicotine administration. Using microtip catheterization, functional parameters of the heart were assessed 12 or 24 hours after infliction of burn trauma. *Results*. Burn trauma led to significantly decreased blood pressure (BP) values whereas nicotine administration normalized BP. As expected, burn trauma also induced a significant deterioration of myocardial contractility and relaxation parameters. After application of nicotine these adverse effects were attenuated. *Conclusion*. The present study showed that transdermal nicotine administration has normalizing effects on burn-induced myocardial dysfunction parameters. Further research is warranted to gain insight in molecular mechanisms and pathways and to evaluate potential treatment options in humans.

## 1. Introduction

Severe burn injury activates a multitude of immunologic defense mechanisms, one of these being the massive production of proinflammatory mediators [1, 2]. Looking at the organ level, one pivotal consequence of burn trauma is cardiac dysfunction [3, 4]. For its pathogenesis the focus of scientific interest has been on proinflammatory cytokines such as TNF-*α*, IL-1*β*, and IL-6, which have been shown to either alone or in concert exert cardiodepressive effects [1, 3, 5]. Elevated levels of IL-1*β*, IL-6, and TNF-*α* corresponded to increased levels of creatinine kinase [5]. Various proinflammatory cytokines demonstrate peak concentrations 12 to 24 hours after trauma [1].

There is a link between the central nervous system and immunologic mechanisms [6]. The vagus nerve axis of parasympathetic activity represents one of the mechanisms that can induce anti-inflammatory effects. Its activation may contribute to preventing a hyperactivation of immune subsystems and reaction overshoot [7, 8]. Macrophages have been identified to be the key target cells for the parasympathetic anti-inflammatory effect of vagus nerve activation. A reason for this is the vagus nerve innervation of most internal organs [6, 9]. Also in other organs such as the heart, the release of TNF-*α* and IL-1*β* was inhibited directly via stimulation of the vagus nerve [10]. Tracey and colleagues identified this signal transduction pathway and named it “cholinergic anti-inflammatory pathway” because of its primary neurotransmitter acetylcholine [6, 11]. Mechanistic studies have shown that the molecular basis for this pathway is the nicotinergic acetylcholine receptor (nAChR), which is also present in the sympathetic part of the autonomic nervous system [6, 12]. Nicotine, a receptor agonist, can stimulate the parasympathetic anti-inflammatory mechanisms via the nAChR [12, 13]. Nicotine is well known as one of hundreds of components of tobacco smoke [14, 15]. However, it has also been used pharmacologically. Transdermal application and absorption of nicotine have been used as a noninvasive application method in a plethora of studies, verifying its anti-inflammatory potential [16–18]. Additionally nicotine application in experimental animal models of rheumatoid arthritis and autoimmune myocarditis, other pathologies based on an overwhelming inflammation, provided evidence for a potential clinical relevance of the immunomodulatory effect of nicotine [19, 20].

The present experimental study was designed to evaluate whether the transdermal application of nicotine is feasible and to evaluate potential beneficial effects on myocardial function after severe thermal injury.

## 2. Material and Methods

**Figure 1:**
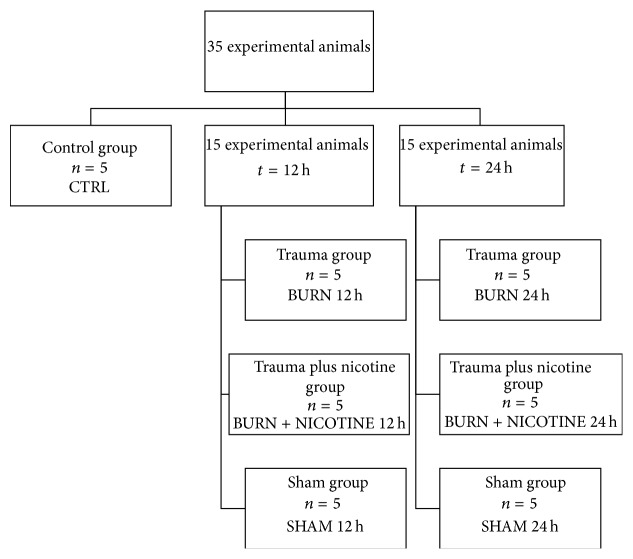
Experimental groups. The classification of 35 experimental animals into seven experimental groups with five experimental animals each is illustrated. Catheter results 12 h and 24 h after experimental burn trauma are compared to control group.

The university committee for the use and protection of animals and the Lower Saxony State Office for Consumer Protection and Food Safety approved the present study (study protocol # 05/1052). We created seven study groups with five animals each. This included one control group (CTRL). Microtip catheterization was done after 12 h or 24 h according to the respective treatment. That resulted in the additional six groups BURN 12 h and BURN 24 h, BURN + NICOTINE 12 h, and BURN + NICOTINE 24 h and SHAM 12 h and SHAM 24 h (Figure 1). The study design, the burn trauma, and nicotine application were previously described [21].

### 2.1. Myocardial Function

**Figure 2:**
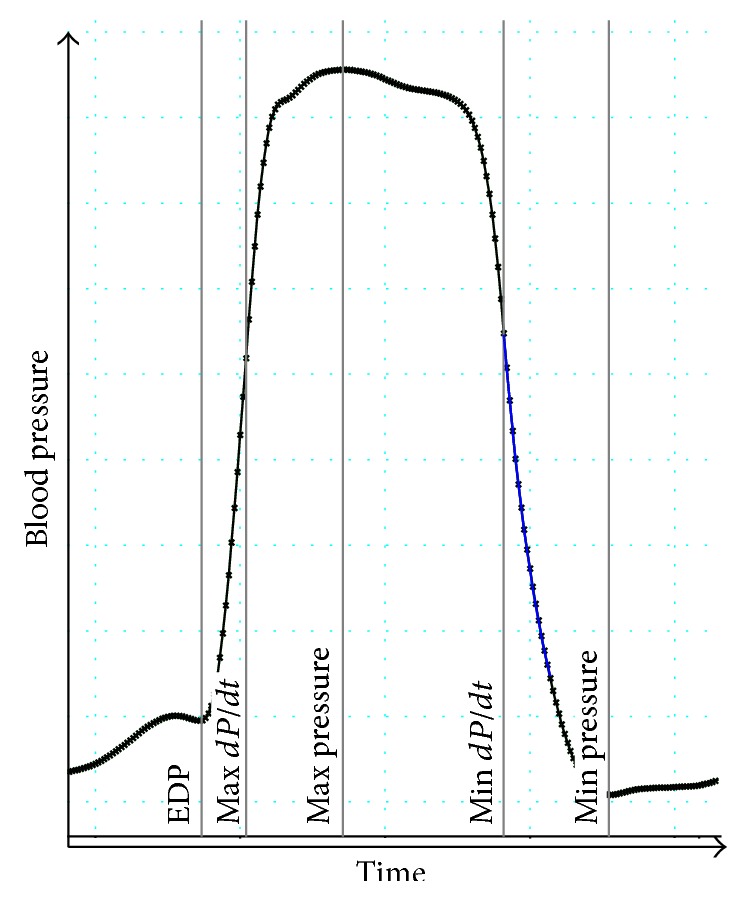
Representative blood pressure curve to illustrate measured parameters. Relevant values are marked. From left to right: end-diastolic pressure (EDP) and maximum blood pressure rise per time (Max *dP*/*dt*), systolic blood pressure (Max pressure) and maximum blood pressure drop per time (Min *dP*/*dt*). Additionally the minimum blood pressure is illustrated (Min pressure).

Microtip catheterization of the left ventricle was carried out in experimental animals of the CTRL group without any previous treatment and in the BURN groups, BURN + NICOTINE groups, and SHAM groups 12 or 24 hours after experimental burn injury or sham trauma, respectively. The right carotid artery was surgically exposed by midline sternotomy. Using microsurgical techniques, a vascular incision large enough to induce the microtip catheter was created (Fa. Millar Instruments, Houston, TX, USA). The correct catheter position was confirmed by the appearance of the characteristic pressure curves and fluoroscopy imaging (Figure 2). Cardiac actions were then recorded for 5 minutes using commercially available analysis software (Chart 5, AD Instruments GmbH, Spechbach, Germany).

### 2.2. Statistical Analysis

We used Prism 5 software for statistical analysis (GraphPad Inc., La Jolla, CA) performing Analysis of variance (ANOVA) followed by Tukey's post hoc test. Statistical significance was set at *p* < 0.05. The results are illustrated as means ± standard deviation (SD).

## 3. Results

### 3.1. Blood Pressure

The results of the microtip catheter for systolic and end-diastolic blood pressure measured 12 and 24 hours after burn trauma showed significantly decreased values for the BURN groups compared to the respective CTRL groups. For both physiological parameters analyzed, nicotine administration increased BP levels significantly compared to the untreated BURN groups except for end-diastolic blood pressure after 24 h. However, the baseline BP value of the control group was not completely reestablished by nicotine application.

**Figure 3:**
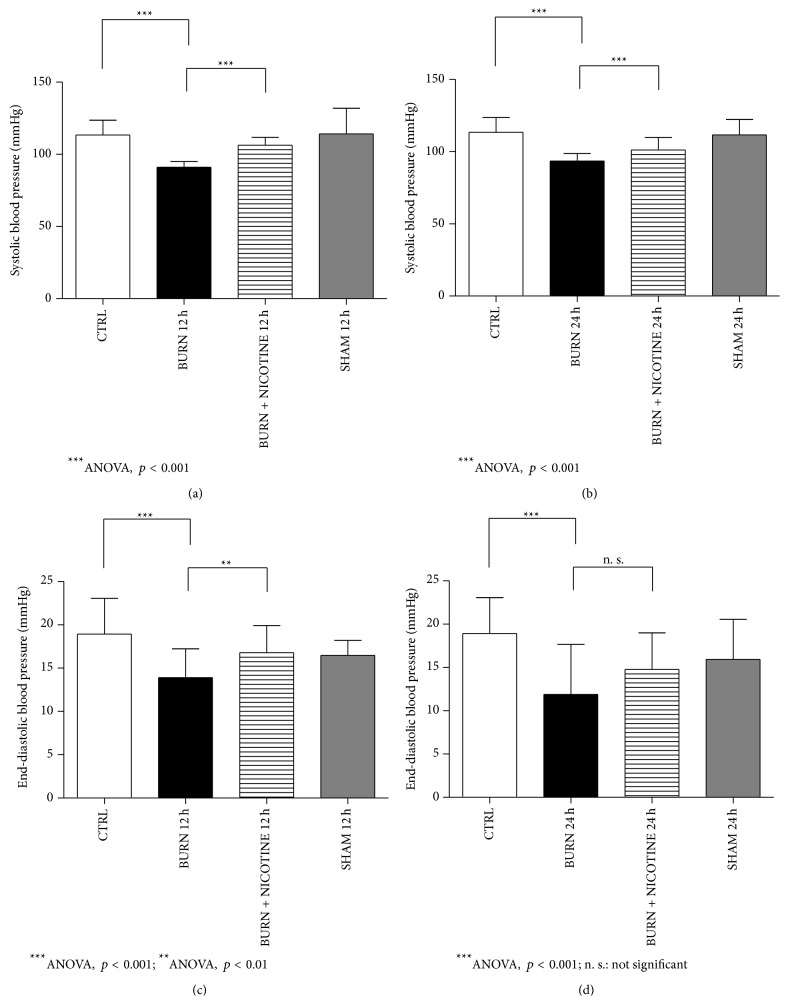
Blood pressure parameters. (a) and (b): systolic blood pressure. (c) and (d): end-diastolic blood pressure. Concerning the experimental design, note decreased BP levels in the BURN groups in contrast to the CTRL group (*p* ≤ 0.05). Transdermal nicotine application at least partially restored this effect. BP values are expressed as means ± SD. Each bar represents *n* = 5 experiments.

After time *t* = 12 h post burn injury, the mean values of the BURN group for the systolic blood pressure were significantly decreased compared with values measured in the CTRL group (Figure 3(a), 91.1 ± 3.8 mmHg versus 113.4 ± 10.3 mmHg, black versus white bar, *p* < 0.001). Nicotine partially normalized the effect of the burn trauma and lead to a systolic blood pressure of 106.3 ± 5.5 mmHg (Figure 3(a), striped bar, *p* < 0.001). The same trend was found at *t* = 24 h after experimental burn procedure. The average systolic BP level of BURN 24 h group of 93.6 ± 5.1 mmHg was restored by nicotine application to 101.2 ± 8.6 mmHg (Figure 3(b), black versus striped bar, *p* < 0.001).

The results of the end-diastolic blood pressure are shown in Figures 3(c) and 3(d). Rats subjected to burn trauma developed significantly lower end-diastolic blood pressure than their CTRL counterparts (*p* < 0.001). The nicotine administration resulted in a significantly higher end-diastolic pressure 12 h after burn trauma whereas the difference after 24 h was statistically not relevant (Figures 3(c) and 3(d), striped versus black bars, *p* < 0.001 after 12 h and not significant after 24 h).

### 3.2. Systole

For the assessment of cardiac function during systole the duration of systole and contractility were measured in terms of maximum blood pressure rise per second.

**Figure 4:**
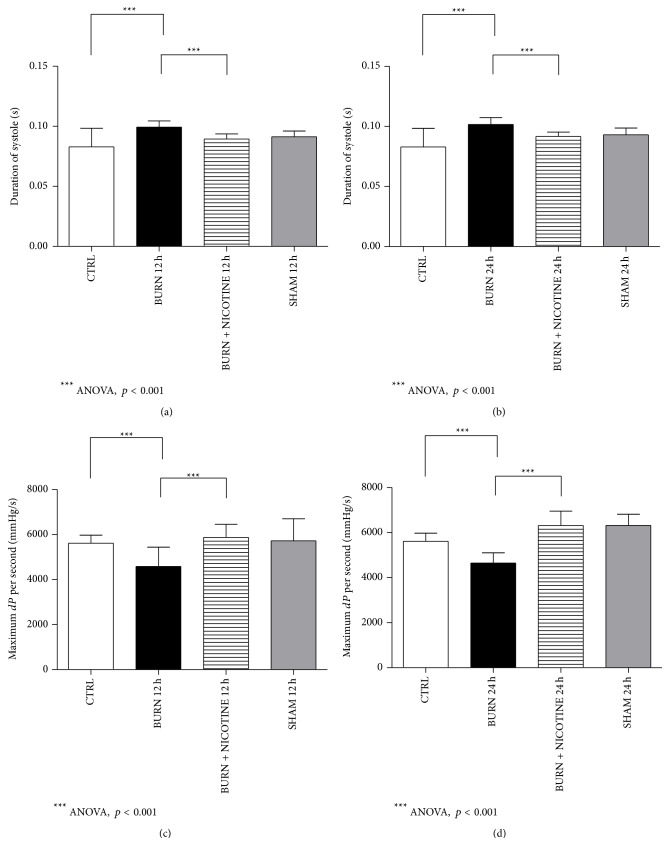
Systole. (a) and (b): duration of systole. The systole duration increased in experimental burn group (black versus white bar, *p* < 0.05). The BURN + NICOTINE groups revealed significantly lower duration of systole values than their burn alone counterparts (striped versus black bar, *p* < 0.05). (c) and (d): maximum increase of blood pressure per second (contractility). Reduced contractility was found for the BURN group (black versus white bar, *p* < 0.05). Nicotine administration showed restored contractility (striped versus black bar, *p* < 0.05). All values are expressed as mean + SD. Each bar represents *n* = 5 experiments.

The duration of the systole was on average 0.083 s in CTRL group (Figures 3(a) and 3(b), white bar). For duration of systole of BURN groups we found mean values of 0.096 s after 12 h and 0.098 s after 24 h, whereas the values of BURN + NICOTINE groups were significantly decreased (Figures 4(a) and 4(b), black versus striped bars, *p* < 0.001).

A significant increase of maximum blood pressure rise per second was observed when results of BURN groups were compared to BURN + NICOTINE groups (Figures 4(c) and 4(d), black versus striped bars, *p* < 0.001). Interestingly, SHAM and BURN + NICOTINE groups displayed slightly higher values for cardiac contractility than CTRL animals (Figures 4(c) and 4(d), grey and striped bars versus white bar). The BURN 12 h group demonstrated a value for maximum blood pressure rise per seconds of 4586 ± 859.7 mmHg/s and the BURN 24 h remained at a low level with 4653 ± 451.4 mmHg/s. In contrast, the BURN + NICOTINE groups revealed 5878 ± 576.3 mmHg/s and 6316 ± 649.9 mmHg/s, respectively, (Figures 4(c) and 4(d), striped bars versus black bars, *p* < 0.05).

### 3.3. Diastole

Based on the assessment of cardiac function during systole we determined the duration of diastole and the maximum blood pressure drop per second (relaxation) for the assessment of cardiac function during the diastolic phase of the cardiac cycle. With regard to the relaxation it is important that a higher negative value indicates a faster relaxation.

**Figure 5:**
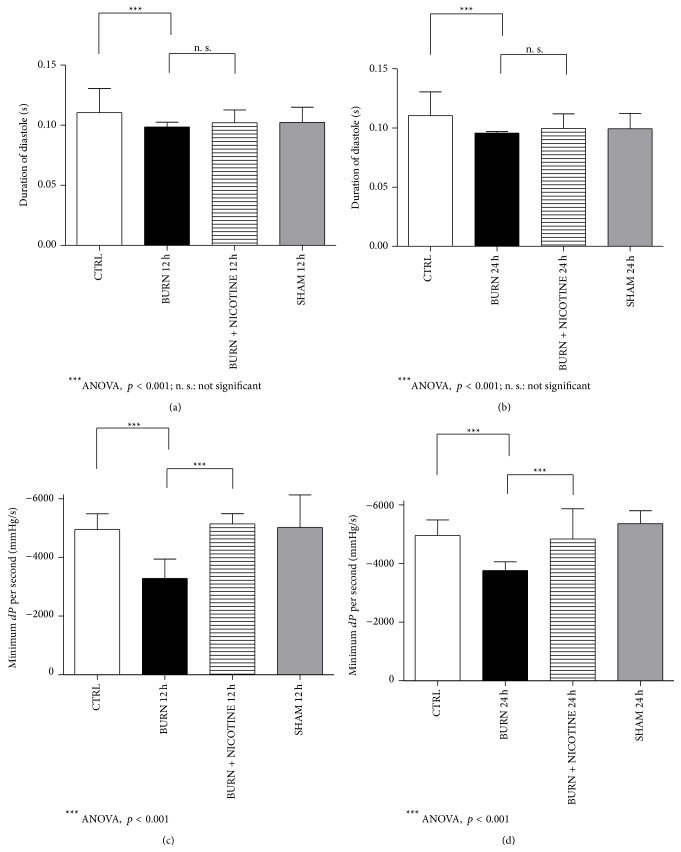
Diastole. (a) and (b): duration of diastole. The BURN group revealed significantly shorter diastole duration than the CTRL group (black versus white bar, *p* < 0.05). After posttraumatic nicotine application this effect is slightly reduced although differences were not statistically relevant (striped versus black bar,* not significant*). (c) and (d): minimum blood pressure difference per second (relaxation). Note that the scale is negative. The difference between the BURN and BURN + NICOTINE animals is marked (black versus striped bars, *p* < 0.05). Interestingly, both SHAM and BURN + NICOTINE groups display higher values than the control group. All results are expressed as mean + SD. Each bar represents *n* = 5 experiments.

The duration of diastole after burn injury was on average 0.96 after 24 h and 0.99 s after 12 h (Figures 5(a) and 5(b), black bars). When burn-injured animals underwent nicotine treatment, duration of diastole was slightly increased compared to burn alone animals (Figures 5(a) and 5(b), striped versus black bars, not significant).

Animals subjected to burn trauma showed a markedly slower relaxation compared to their SHAM and CTRL counterparts (Figures 5(c) and 5(d), black versus grey and white bars, *p* < 0.001). Nicotine treatment induced a relaxation similar to sham animals (Figures 5(c) and 5(d), black versus striped bars, *p* < 0.001).

### 3.4. Heart Rate

**Figure 6:**
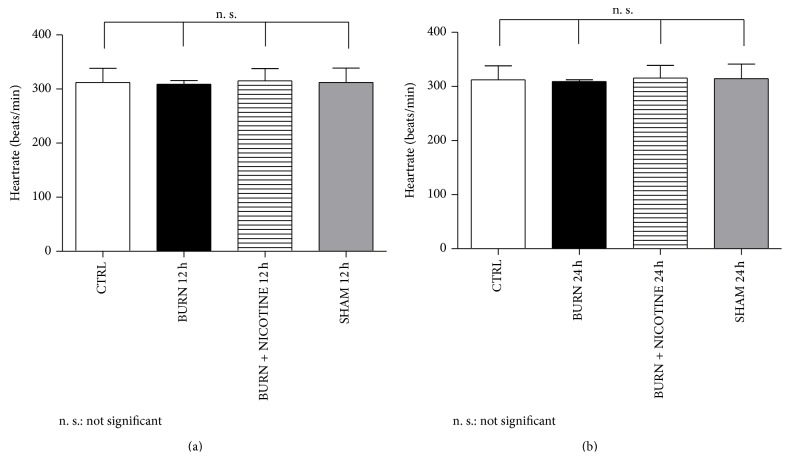
Heart rate. (a) and (b): heart rate. In the analysis of heart rate, no significant differences between the groups of experimental animals were found. The values are expressed as mean + SD. Each bar represents *n* = 5 experiments.

Concerning the heart rate no significant differences were found 12 and 24 h after burn injury. The heart rate values ranged from 309.3 ± 3.2 beats/min (Figure 6(b), black bar) up to 315.4 ± 23.4 beats/min (Figure 6(b), striped bar).

## 4. Discussion

Intense burn trauma imposes life-threatening consequences to the victim. The multifactorial pathogenesis of burn-induced cardiac dysfunction has been extensively evaluated and described. This study was conducted to evaluate whether the known immunomodulatory effect of nicotine can improve cardiac function after burn trauma.

Previous experiments of our research group were looking at direct electrical stimulation of the vagus nerve and we could prove attenuation of proinflammatory cytokine production after electrostimulation of the parasympathetic axis [10, 22]. Additionally several studies used transdermal nicotine application to stimulate the parasympathetic axis because nicotine administration has been shown to induce anti-inflammatory effects [16, 17, 21, 23]. Clinically, this has been demonstrated in patients suffering from colitis ulcerosa [17, 23]. But in contrast, nicotine has adverse effect on Crohn's disease [23]. The reason for this ambiguous reaction in inflammatory bowel diseases is still not understood in entirety [23]. Still nicotine itself is seen doubtfully as an additive aspect in the treatment after burn trauma especially due to described adverse effects on wound healing [24, 25]. This points up the importance of further experiments to distinguish the positive anti-inflammatory effects of nicotine from adverse local effects on wound healing.

The present study showed that the administration of nicotine partially restores or normalizes decreased systolic blood pressure following burn injury. The results published by the groups of Sambol and Adams also revealed decreased systolic blood pressure at 24 hours after burn trauma [4, 26]. However decreasing the burn-induced increased activity of cardiodepressive cytokines like TNF-*α* and IL-1 thereby leading to normalization of blood pressure after a burn trauma [4, 26]. Thus, there is a negative correlation between circulating proinflammatory cytokine concentrations of TNF-*α* and IL-1 and the systolic blood pressure [5].

The left ventricular systolic blood pressure has a direct relationship to the arterial blood pressure that is based on the intravascular blood volume, stroke volume, and the total peripheral resistance. Burn trauma affects all of these three parameters. Capillary leakage results in severe fluid loss and decreased intravascular blood volume; the decreased contractility of the ventricle generates reduced stroke volume and circulating proinflammatory cytokines such as IL-1*β* result in total peripheral resistance loss [3, 27]. Nicotine effectively reduces the concentration of vasodilatory cytokines such as IL-1*β* and thereby leads to an increased total peripheral resistance [12]. In addition, the concentrations of TNF-*α*, IL-1*β*, and IL-6 are reduced which diminishes and, at best, abolishes their negative impact on cardiac contractility [3]. Stimulation of sympathetic neurons via nicotine application might support the restoration of systolic blood pressure. Additionally the volume resuscitation after burn trauma could contribute to the normalization of systolic blood pressure levels. However, sole fluid therapy is insufficient to abolish burn-induced cardiac dysfunction [28], as also seen in our BURN group.

For end-diastolic blood pressure levels burn injury also resulted in decreased levels. This parameter is essentially determined by the intravascular volume and contractile force of the left ventricle. So it may be deduced that the increase of the end-diastolic blood pressure of BURN + NICOTINE groups compared to BURN groups is a result of an impact on left ventricular function. This would be contradictory to the measurements of the other parameters of myocardial function in the present study, indicating a positive effect of nicotine administration on left ventricular function. A second explanation for the increase in end-diastolic blood pressure according to nicotine application is based on the posttraumatic intravascular volume. The large burn-induced volume loss followed to the capillary leak results in a decreased intravascular volume and consecutively in a decreased end-diastolic blood pressure. In this context, Ipaktchi et al. were able to show that immunosuppressive therapy after burn trauma reduces the capillary leakage significantly [29]. This group used the specific inhibition of mitogen-activated protein kinases that has been shown to be a signal transduction element of the proinflammatory signaling cascade. This pathway is also inhibited by nicotine, which was shown by Oke and Tracey [30]. The improved end-diastolic blood pressure may result secondarily via the Frank-Starling mechanism.

The BURN groups showed significantly higher values for duration of systole compared to the control group. This was accompanied by significantly decreased values for the maximum blood pressure rise per second and a shorter duration of diastole. Previous studies also revealed a burn-induced contractile dysfunction [4, 26]. Additionally burn trauma affected diastolic cardiac function. Again, nicotine could attenuate the burn-induced negative effect: The BURN + NICOTINE and SHAM groups revealed increased levels compared to the BURN group and even to control group. Comparative data for this purpose does merely exist to our knowledge. Only the work of Adams and colleagues described the same effect of burn trauma [4]. Their and our results reveal that thermal trauma leads to systolic and diastolic cardiac dysfunction. As a result of our experiments, the application of nicotine has demonstrated, with few exceptions without significant differences, a positive effect on systolic and diastolic function after burn trauma.

Heart rate showed no significant differences. This is consistent with the results of several authors [5, 10, 26, 31]. Adams and colleagues found a steady heart rate in a burn model 24 h after trauma [4]. Similarly, Sambol, Maass, and our own results of burn models showed no changes in heart rate [5, 10, 26]. It is noteworthy that nicotine administration does not induce bradycardia. Bradycardia would be expected from stimulation of the vagal nerve. Interestingly however, in the literature there are data that nicotine has a more tachycardic effect [15, 17]. However, an additional effect of nicotine on sympathetic neurons cannot be excluded and could be an explanation for this aspect.

## 5. Conclusions

In the present study, we found that in a rat model of severe burn injury the trauma causes decreased blood pressure and a decrease in contraction and relaxation velocity of the heart. Adding on to the current knowledge base, further experiments will be done to explore the potent anti-inflammatory mechanisms of activation of the parasympathetic neural system. Our encouraging results that led to restoration of nearly normal myocardial function parameters have already prompted us to follow up on this path.

Whether nicotine may also affect the lethality of severe burn trauma was not the subject of these investigations. This would be of great importance for further studies.
